# Diagnostic and predictive value of Doppler ultrasound for evaluation of the brain circulation in preterm infants: a systematic review

**DOI:** 10.1038/s41390-020-0777-x

**Published:** 2020-03-26

**Authors:** Fleur A. Camfferman, Robbin de Goederen, Paul Govaert, Jeroen Dudink, Frank van Bel, Adelina Pellicer, Filip Cools, eurUS.brain group, Thais Agut, Ana Alarcon, Roberta Arena, Marco Bartocci, Mayka Bravo, Fernando Cabañas, Nuria Carreras, Olivier Claris, Jeroen Dudink, Monica Fumagalli, Paul Govaert, Sandra Horsch, Alessandro Parodi, Adelina Pellicer, Luca Ramenghi, Charles C. Roehr, Sylke Steggerda, Eva Valverde

**Affiliations:** 1 Department of Neonatology, Universitair Ziekenhuis Brussel, Vrije Universiteit Brussel, Brussels, Belgium; 2 Dutch Craniofacial Centre Rotterdam, Department of Plastic and Reconstructive Surgery, Erasmus Medical Center University, Rotterdam, The Netherlands; 3 Department of Neonatology, Erasmus Medical Center University, Sophia Children’s Hospital, Rotterdam, The Netherlands; 4 Department of Neonatology, ZNA Middelheim, Antwerp, Belgium; 5 Department of Rehabilitation and Physical Therapy, Gent University Hospital, Gent, Belgium; 6 Department of Neonatology, University Medical Center Utrecht, Wilhelmina Children’s Hospital, Utrecht, The Netherlands; 7 Department of Neonatology, La Paz University Hospital, Madrid, Spain; 8 Department of Neonatology, Institut de Recerca Pediàtrica, Hospital Sant Joan de Déu, Barcelona, Spain; 9 Catholic University of the Sacred Heart, A. Gemelli Hospital, Rome, Italy; 10 Department of Women’s and Children’s Health, Karolinska University Hospital, Karolinska Insitute, Stockholm, Sweden; 11 Department of Neonatology, Quironsalud Madrid University Hospital and Biomedical Research Foundation, La Paz University Hospital Madrid, Madrid, Spain; 12 Service de néonatologie et de réanimation néonatale, Hospices Civils de Lyon, Université Claude Bernard Lyon, Lyon, France; 13 Department of Clinical Sciences and Community Health, University of Milan, Milan, Italy; 14 Fondazione IRCCS Ca’ Granda Ospedale Maggiore Policlinico NICU, Milan, Italy; 15 Department of Neonatology, Helios Klinikum Berlin Buch, Berlin, Germany; 16 Department Clinical Science Intervention and Technology (CLINTEC), Karolinska Institutet, Stockholm, Sweden; 17 Neonatal Intensive Care Unit, Istituto Giannina Gaslini, Via Gaslini 5, 16148 Genoa, Italy; 18 Department of Paediatrics, Medical Sciences Division, Newborn Services, University of Oxford, Oxford, UK; 19 Department of Neonatology, Leiden University Medical Center, Leiden, The Netherlands

## Abstract

**Introduction:**

Very and extremely preterm infants frequently have brain injury-related long-term neurodevelopmental problems. Altered perfusion, for example, seen in the context of a hemodynamically significant patent ductus arteriosus (PDA), has been linked to injury of the immature brain. However, a direct relation with outcome has not been reviewed systematically.

**Methods:**

A systematic review was conducted to provide an overview of the value of different cerebral arterial blood flow parameters assessed by Doppler ultrasound, in relation to brain injury, to predict long-term neurodevelopmental outcome in preterm infants.

**Results:**

In total, 23 studies were included. Because of heterogeneity of studies, a meta-analysis of results was not possible. All included studies on resistance index (RI) showed significantly higher values in subjects with a hemodynamically significant PDA. However, absolute differences in RI values were small. Studies using Doppler parameters to predict brain injury and long-term neurodevelopmental outcome were inconsistent.

**Discussion:**

There is no clear evidence to support the routine determination of RI or other Doppler parameters in the cerebral arteries to predict brain injury and long-term neurodevelopmental outcome in the preterm infant. However, there is evidence that elevated RI can point to the presence of a hemodynamically significant PDA.

## Introduction

Acquired brain injury is common in the perinatal period. Many extremely and very preterm infants have injury-related long-term neurodevelopmental problems.^1^ Extremes and fluctuations in brain perfusion have been linked to germinal matrix hemorrhage, intraventricular hemorrhage (IVH), and white matter injury.^2–5^ In the adult brain, perfusion is controlled through cerebral autoregulation, the intrinsic capacity of cerebral vessels to adjust muscle tone in order to keep flow more or less constant over a range of blood pressures.^6^ Several studies demonstrated that cerebral autoregulation might not be fully developed in preterm infants, especially in the lower range of gestational ages. Consequently, extremes of systemic perfusion are transmitted unaltered to brain tissue.^7^ Low systemic blood pressure is thought to play an etiological role in the development of IVH in preterm infants.^8–10^ There are studies showing that maintaining blood pressure within normal ranges can prevent severe IVH.^5^ However, there are no clear data demonstrating that this would improve long-term outcome in preterm infants.^11,12^ Low systemic blood flow is associated with IVH with impaired cerebral autoregulation.^13^ In addition, impaired autoregulation is correlated with adverse outcome in infants.^14,15^

In 1979, Bada et al. first reported that cerebral Doppler studies played a role in the diagnosis of neonatal intracranial injury.^16,17^ Doppler provides a valuable tool to follow changes in flow velocities, to assess changes in cerebrovascular resistance and to determine the lower limits of cerebral blood flow autoregulation.^18,19^ For quantitative measurement of flow in a specific vessel, however, knowledge of the internal vascular diameter is necessary, which has proven to be inaccurate in small vessels.^20^ In addition, the angle of insonation between the vessel and the Doppler ultrasound beam has to be as small as possible to prevent inaccuracy in calculating the Doppler shift. To bypass these challenges, many different indices were developed to approximate flow velocity as an estimate of flow, like pulsatility index (PI) and the resistance index (RI). However, the relation of Doppler indices in preterm infants with brain injury and outcome remains controversial. In term birth asphyxia, for instance, low RI in the anterior cerebral artery has been associated with an adverse neurodevelopmental outcome,^21^ although a relation with extent of specific regional injury on magnetic resonance imaging (MRI) is not documented. In other pathologies, like hydrocephalus, the role of Doppler ultrasound in diagnosis and prognosis is less clear.^22,23^

In the present systematic review, we study the value of various Doppler-derived variables in cerebral arteries to predict intracranial injury and long-term neurodevelopmental outcome in the preterm infant. Our search also focused on the relation between cerebral arterial Doppler parameters and patent ductus arteriosus (PDA), since a hemodynamically significant PDA is thought to be one of the determinants of altered brain perfusion patterns in the preterm infant.

## Methods

### Search strategy

Electronic literature searches were performed in MEDLINE, EMBASE, and the Cochrane Central register of Controlled Trials. Full MEDLINE search was first performed on 08-07-2015 using the following Mesh terms: “Infant, Newborn”, “Echoencephalography”, “Ultrasonography, Doppler”, “Blood Circulation Time”, “Brain”, “Blood Vessels”, “Microcirculation”, “Cerebral Arteries”, “Cerebral Veins”, “Blood Flow Velocity”, and “Cerebrovascular Circulation”. Appropriate text words were added as well. For the full MEDLINE search, see Table 1. Search was restricted to human studies. There was no restriction in publication date. The search was double-checked by the Erasmus University Rotterdam librarian. A weekly current awareness alert in MEDLINE was set, and last update of the search was performed April 2018. In Cochrane, last search was performed April 2018; last EMBASE search was performed may 2015. We looked for additional studies in the reference lists of the studies identified. In addition, we screened some reference suggestions provided by two of the authors, both experts in the field of cranial ultrasound in the newborn (P.G. and A.P.).

**Table 1:** Full search strategy in MEDLINE.

Time	Full search performed on 08-07-2015
Items	(Neonate* [tiab] OR Newborn* [tiab] OR Preterm* [tiab] OR Premature* [tiab]) OR “Infant, Newborn”[Mesh]
	And((((((“Echoencephalography”[Mesh]) OR “Ultrasonography, Doppler”[Mesh]) OR “Blood Circulation Time”[Mesh]) OR (((Ultrasound [tiab] OR US [tiab] OR echo [tiab] OR Doppler [tiab]) AND (Brain [tiab] OR Cerebral [tiab] OR Cranial [tiab])))) OR ((Index [tiab] AND (Resistive [tiab] OR Resistance [tiab] OR Pourcelot [tiab])))) OR “velocity time” [tiab]) OR velocity OR Echoencephalograp* [tiab] OR Ultrasonogra* [tiab]
	and((((cerebral [tiab] OR brain [tiab] OR cranial [tiab]) AND (flow [tiab] OR blood flow [tiab] circulation [tiab])))) OR ((“Brain”[Mesh] AND (“Blood Vessels” [Mesh] OR “Microcirculation”[Mesh])) OR “Cerebral Arteries”[Mesh] OR “Cerebral Veins”[Mesh] OR “Blood Flow Velocity”[Mesh] OR “Cerebrovascular Circulation”[Mesh]).
Result	Result: 4069 titles

### Study selection

All experimental as well as observational studies in which Doppler technique was used to assess cerebral perfusion in preterm infants (gestational age <37 weeks) and related to mortality and morbidity (IVH, periventricular leukomalacia (PVL), and PDA) during the intensive case period or related to long-term morbidity were included. For observational studies, both prospective cohort and case–control studies were eligible for inclusion. Case reports and case series, defined as ≤10 patients in the study and/or ≤5 patients per outcome group, as well as narrative reviews were excluded. Also, studies focusing on intervention (e.g., cerebral blood flow before and after duct ligation), only describing normative values of a cerebral Doppler index, or in which the main subject was asphyxia or hydrocephalus (not meeting the review question) were not considered eligible for inclusion. Only articles in English, German, French, or Dutch were included. Two review authors (P.G. and F.A.C.) independently assessed eligibility for inclusion of the identified studies based on abstract. Any disagreement was solved through discussion. Subsequently, R.d.G. and F.A.C. independently performed assessment of full-text articles. Disagreement was solved through discussion.

### Data collection and data extraction

**Fig. 1:**
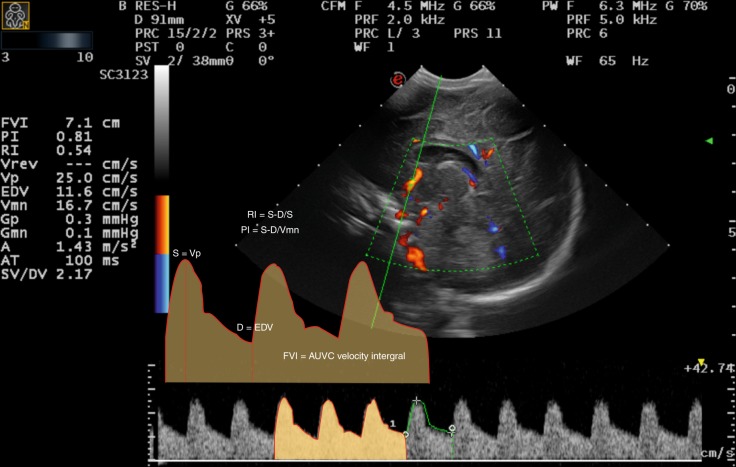
GMH/IVH: Doppler systematic review: arterial flow indices. AUVC area under the velocity curve, FVI flow velocity integral, Vmn mean velocity = MV in the text.

The methodology for data collection and analysis was based on the Cochrane Handbook of Systematic Reviews of Interventions.^24^ Data extraction was performed independently by two authors (R.d.G. and F.A.C.) using a pre-designed data collection form. Discrepancies in data extraction were resolved by discussion. This extraction form included (I) information on Doppler technique, insonated vessel(s), and Doppler parameter(s) (i.e., RI, PI, mean velocity, end-diastolic velocity, peak systolic velocity, time averaged velocity, etc.); (II) basic patient characteristics; (III) correlation with systemic parameters; (IV) correlation with pathology during neonatal intensive care unit (NICU) stay; and (V) correlation with long-term outcome. For clarity purposes, we used consistent terminology when describing Doppler indices. RI, also called Pourcelot index or resistency index, was used for the index described by the following equation: ${\rm{RI}} = \frac{{{\rm{Vs}} - {\rm{Vd}}}}{{{\rm{Vs}}}}.$ In this equation, Vs is the systolic velocity in cm/s and Vd is the diastolic velocity in cm/s. PI was used for the index described by the function: ${\rm{PI}} = \frac{{{\rm{Vs}} - {\rm{Vd}}}}{{{\rm{MV}}}}.$ In this function, Vs is the systolic velocity in cm/s, Vd is the diastolic velocity in cm/s, and MV is the mean velocity in cm/s. The relevant Doppler parameters used in this review are explained in Table 2 and Fig. 1. Where possible, 95% confidence intervals of the Doppler-derived parameters were calculated, using sample size, standard deviation, and sample mean.

**Table 2:** Different Doppler parameters and explanation.

Abbreviation	Full term	Equation/explanation	Synonym
RI	Resistance index	Vs − Vd/Vs	Pourcelot index Resistency index
PI	Pulsatility index	Vs − Vd/MV	
MV	Mean velocity	Mean velocity calculated over a series of cardiac cycles	
Vps	Peak-systolic velocity	Highest velocity in the cardiac cycle	Vs
Ves	End-systolic velocity	Velocity at the end of the systolic phase of cardiac cycle	
Ved	End-diastolic velocity	Lowest velocity in the cardiac cycle	Minimum CBFV Vd
AUVC	Area under the velocity curve	Represents mean flow velocity	
CBFF	Cerebral blood flow fluctuation	Interquartile range of velocity	
Min–max MV ratio	Ratio of minimum and maximum MV	Minimal MV/maximal MV	
Vmean ratio	Ratio of VM at different times	MV in first 12 h/MV at 12–168 h	
TAV	Time averaged velocity	Vmax/2	
CV%	Coefficient of variability	Coefficient of variation of AUVC values of 20 consecutive cardiac cycles	
BFV	Blood flow velocity		
	Cerebrovascular perfusion pressure	Mean BFV/(mean BFV − diastolic BFV) × (mean BP − diastolic BP)	
	Cerebral blood flow resistance	Cerebral perfusion pressure/RI

### Assessment of methodological quality

Three authors (F.C., R.d.G., and F.A.C.) independently assessed the risk of bias. For cohort studies and case–control studies, the appropriate checklist from the Scottish Intercollegiate Guidelines Network (SIGN) was used (http://www.sign.ac.uk/checklists-and-notes.html). Any disagreement was resolved through discussion. Overall quality of the included studies was defined as “low risk of bias,” “unclear risk of bias,” or “high risk of bias”. Decisive items for this overall quality were study group selection, blinding, and correction for confounders.

### Statistics

Because of heterogeneity of Doppler indices, insonated blood vessels, and outcome measures, a quantitative analysis of included studies could not be performed. A narrative summary of the included studies has been provided instead.

## Results

### Study selection

**Fig. 2:**
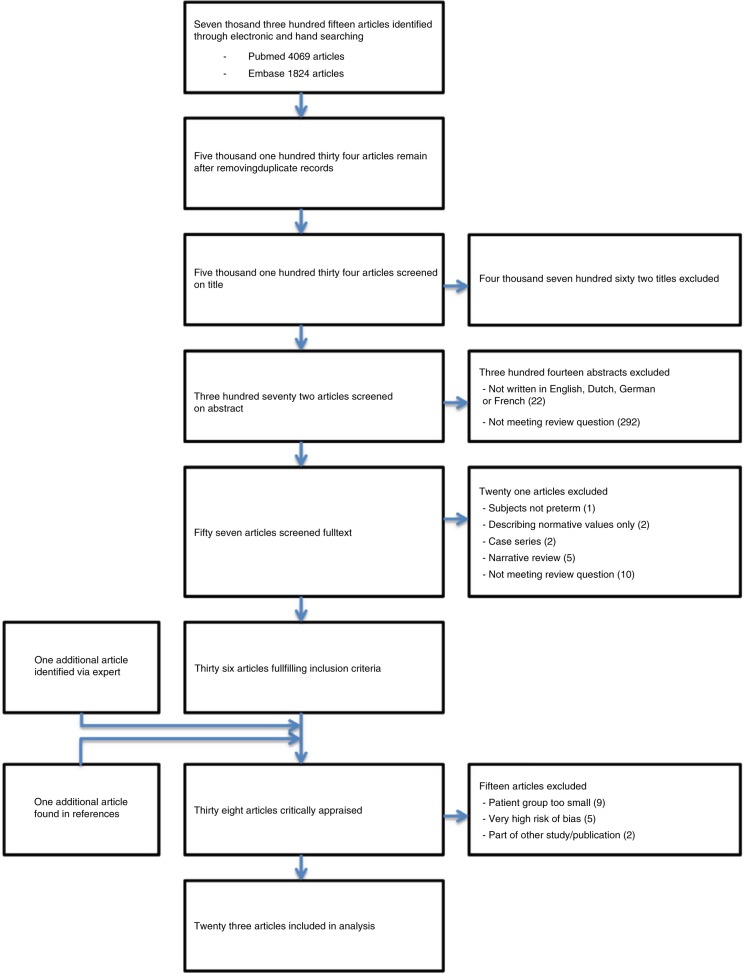
GMH/IVH: Doppler systematic review.

The full search identified 7315 articles potentially relevant to our research question, of which 5134 remained after removal of the duplicates. There were no Cochrane reviews meeting our review question. After screening on title and abstract, 57 articles remained. After reading full text, eventually 36 articles met inclusion criteria. One additional study was identified via an expert in the field and one article was identified searching the references of the included studies. Finally, 38 articles were critically appraised, which further led to exclusion of 15 articles based on very high risk of bias, very small subgroups (<5 cases per outcome group), or overlapping population with other references. The remaining 23 articles were included in this review (refs.; ^25–47^ see Fig. 2 and Appendices 1 and 2).

### Study characteristics

In total, 23 studies were included with a total of 2095 preterm participants (median 58 participants per study, range 18–452). Characteristics of the included studies are shown in Appendix 1. In seven older studies,^25,27,28,30–33^ the continuous wave technique with a duplex flowmeter was used to measure Doppler indices. In the remaining 16 studies, real-time two-dimensional (2D) gray-scale ultrasonography combined with Color Doppler was applied.

### Quality assessment

**Fig. 3:**
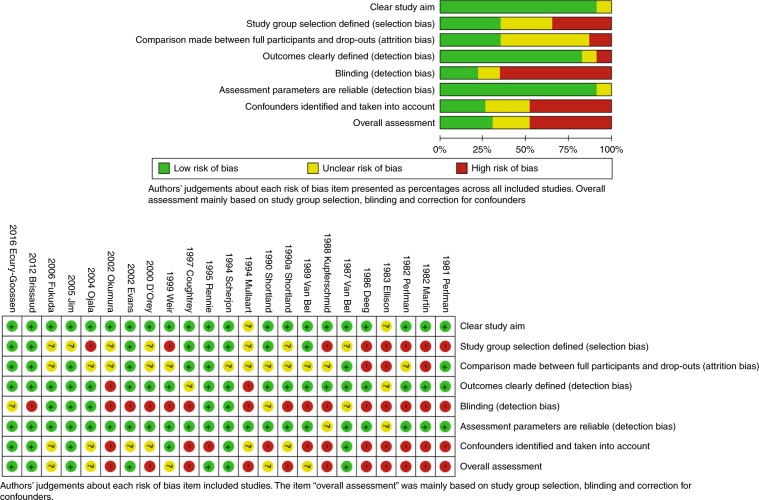
GMH/IVH: Doppler systematic review: bias results.

Out of the 23 included articles, 18 met cohort study design criteria, 2 were considered to be convenience samples with a cohort design, and 3 met case control study criteria. After quality assessment, 7 studies (30%) were considered to have a low risk of bias, 5 (22%) were considered to have an unclear risk of bias, and 11 (48%) were considered to have a high risk of bias. Considering the separate items of the SIGN checklist, the quality of reporting of the study aim, the definition of outcomes, and the definition of assessment parameters were considered to be good in most of the studies. Overall, criteria for blinding and correction for confounders were less frequently met and therefore a potential risk of bias existed (see Fig. 3).

### Results of individual studies

In Appendix 2, a summary of the results of each study is presented.

#### Persistent ductus arteriosus

Eleven studies examined the relationship between cerebral blood flow (CBFV) and PDA. In 9 (82%) studies, the Doppler parameter to approach CBFV was RI. All studies investigating the RI in the anterior cerebral artery (ACA—8 studies), of which 6 studies considered as high risk of bias and 2 studies considered as low risk of bias, found a significantly higher RI in hemodynamically significant PDA. Mean RI varied between 0.78 and 1.2 in a large PDA compared to RI of 0.61–0.81 in preterm infants without a significant PDA.^25,26,28,29,31,40,44,47^ Five of these studies used the duplex method without 2D ultrasound. RI in the middle cerebral artery (MCA) was investigated in two studies,^39,40^ showing a significantly higher RI in patients with PDA. These studies were considered to be moderate and high risk of bias studies, respectively. The internal carotid artery (ICA) was examined in two studies.^40,47^ Both concluded that there was a significantly higher RI in the ICA in the presence of a hemodynamically significant PDA (median RI 0.82 in PDA versus 0.75 in controls—*p* < 0.001^47^ and mean RI 0.97 in PDA versus 0.81 in controls—*p* < 0.05^40^). The RI in the common carotid artery (CCA) and in the basilar artery and striatal and pial arteries did not show significant differences in preterm infants with and without hemodynamically significant PDA.^28,47^ Other parameters studied in relation to PDA were coefficient of variation,^38^ PI,^39^ MV,^28,34,39,40^ peak-systolic velocity, end-systolic and end-diastolic velocities,^29^ or maximum and minimum velocities (ratio min–max MV).^34^ For MV, three out of four studies showed significantly lower values in infants with hemodynamically significant PDA. However, absolute MV values differed substantially between those studies (e.g., MV in the ACA ranged from 4.0 to 30.2 cm/s in infants with PDA and from 6.5 to 34.9 cm/s in controls). Quality assessment of these studies classified two of these studies to be of high risk of bias^28,40^ and one of moderate risk of bias.^39^

#### Doppler indices and the occurrence of IVH and PVL

In nine studies, a relation between the occurrence of intracranial hemorrhage and/or white matter injury and CBFV was investigated. Six studies examined the relation between CBFV and periventricular hemorrhage, IVH, or peri-intraventricular hemorrhage (PIVH). In five articles (three low risk of bias studies and two high risk of bias studies), no significant correlation was reported using peak-systolic velocity, RI, PI, MV, or cerebral blood flow fluctuation as Doppler parameters for CBFV.^33,35,36,41,46^ One low risk of bias study concluded that RI was significantly lower and the area under the velocity curve (AUVC), RI variation, and AUVC variation in the first week of life were significantly higher in infants who developed severe PIVH.^30^ Two studies explored whether a temporal relation could be detected between an abnormal RI and the onset of IVH. No such relationship could be demonstrated.^27,30^ In five studies, the predictability of PVL by CBFV was assessed. In three of these studies, no correlation was found between CBFV and PVL, using either ACA peak-systolic velocity,^46^ ACA mean velocity,^33^ or MCA mean velocity.^36^ In one study, infants developing PVL had a reduced RI in the ACA in the first 72 h of life,^42^ whereas in another study a lower mean CBFV was not found until beyond the first week of life in the posterior cerebral artery and the ICA and until beyond the second week of life in the ACA, MCA, and basilar artery.^45^

#### Neurodevelopmental outcome

The relation between neonatal CBFV variables and long-term neurodevelopmental outcome was studied in four publications. Different scales were used to evaluate neurodevelopmental outcome.

One moderate risk of bias study showed that low mean CBFV, low CBF resistance (calculated by dividing mean blood pressure by MV as previously described by Evans et al.^48^) and low cerebrovascular perfusion pressure (calculated using an equation previously validated by Aaslid et al.^49^) in the ACA on the first day of life in non-ventilated infants was associated with a lower Griffith’s score at 12 months’ corrected age.^43^ A second study, classified as low risk of bias, failed to demonstrate a correlation between the Griffith’s scale at 18 months’ corrected age and the CBFV in the ACA.^37^ However, infants with an adverse outcome failed to show the steady rise in CBFV during the first days of life, which is observed in infants with normal outcome. One study showed an association between Vmean ratio (MV in the first 12 h/MV at 12–168 h) and the Prechtl score at term-equivalence but could not demonstrate a correlation between MV or the ratio between the minimal and maximal MV and the Touwen score at 6 and 12 months after birth.^36^ The fourth study, classified as moderate risk of bias, used Griffith’s mental developmental scale and Bayley scales of infant development at 2 years’ corrected age.^32^ The authors concluded that the RI measured by the duplex flowmeter in the ACA in the first week of life was significantly higher in infants with major impairment.

## Discussion

Twenty-three studies investigating a variety of flow velocity parameters in relation to the presence of a hemodynamically significant PDA, intracranial abnormalities, or neurodevelopmental outcome at 12–18 months’ corrected age were identified. Main findings of the studies concerning PDA showed that a hemodynamically significant PDA can be predicted by arterial Doppler patterns. However, there was no consistent evidence that any of the various Doppler-derived variables can predict either development of brain injury or long-term neurodevelopmental impairment.

### Persistent ductus arteriosus

This review found an association between a hemodynamically significant PDA and higher RI and lower MV in the ACA, MCA, and ICA. The presence of a PDA has also been associated with adverse outcome^50–52^ and lower cerebral volumes at term-equivalent age.^53^ Altered cerebral blood flow patterns secondary to a PDA, as identified by cerebral Doppler, could therefore help to guide therapeutic strategies. For instance, Bravo et al.^54^ showed that an RI of ≥0.74 measured 24 h after termination of ibuprofen treatment was the best biomarker of moderate-to-large PDA (sensitivity 82% (52–95%), specificity 72% (54–84%), positive predictive value 50% (29–71%), and negative predictive value 92% (75–98%). Interestingly, surgical closure of a PDA did not change RI values^47^ and did not improve the long-term neurodevelopmental outcome.^55^ However, in these studies a hemodynamically significant PDA usually is defined only by PDA size, flow pattern, and signs of pulmonary overflow expressed as left atrium-to-aorta ratio (LA:Ao). Other echographic signs of systemic repercussion, like low superior vena cava (SVC) flow or absent or reverse flow in any post ductal organ, are usually not taken into account. It might be that a large PDA causes lung overflow but that systemic circulation is not compromised because of compensatory mechanisms.

The high RI and low MV in the ACA is thought to be caused by ductal steal, first described by Spach et al. in 1980.^56^ Ductal steal describes the phenomenon of reduced or even reversed end-diastolic flow to organs secondary to the large ductal shunt toward the lungs during diastole. Since the cerebral circulation in the preterm infant is considered to be a low-resistance system in which diastolic blood flow is an important component,^57^ the preterm brain is thought particularly sensitive to this phenomenon. However, other components must play a role as there is not a consistent relationship between PDA and the long-term outcome, and ductal closure does not consistently lead to normalization of RI and MV.^28,47^ Interestingly, the RI in the CCA, basilar artery, striatal and pial arteries did not differ among preterm infants with and without a hemodynamically significant PDA.^28,47^ Ecury-Goosen et al.^47^ speculated that the diameter of the insonated artery influences the RI. In the smaller arterial vessels, the diastolic component of the arterial velocity envelope is proportionally higher than in the larger arteries, leading to lower RI in the first.^58^ This theory only partially explains why in the smaller arteries, such as the striatal and pial arteries, no differences in the RI are found between patients with and without PDA. However, one would expect an effect of PDA in the calculated RI in the larger basilar arteries and CCA.

It is important, however, to realize the difference between statistical significance and clinical relevance. The reported values for RI in the articles included in this review show normative values ranging from 0.61 to 0.81, with values associated with a large PDA ranging from 0.78 to 1.2, therefore showing an overlap. Even in the presence of a hemodynamically significant PDA, the RI is not always consistently altered. One explanation could be that autoregulation is unaltered,^59^ so that the ability to maintain cerebral blood flow stable irrespective of alterations in systemic blood flow remains. Another explanation might be that the RI is not affected when diastolic and systolic flows are equally affected, such as in low cardiac output state. In addition, in some of the included articles, RI values of >1.0 were reported as a result of negative diastolic flow. However, accuracy of these values is debatable, since the systolic/diastolic velocity ratio used to calculate RI approaches infinity and therefore loses meaning.^60^ The majority of authors report RI with a range between 0 and 1 as they only use it when the diastolic flow is not reverse.

### Doppler indices and the occurrence of IVH and PVL

In this review, we did not find a correlation between the arterial cerebral blood flow Doppler-derived variables and documented structural brain damage (IVH and PVL) during the neonatal period. It is possible that measurements were not done frequently enough. Change in cerebrovascular resistance is a dynamic process, therefore non-continuous assessment may lead to misinterpretation. Van Bel et al.^30^ indeed found a correlation between RI, AUVC, and their variability and the development of IVH during the first week of life. Unfortunately, a temporal relationship between Doppler changes and onset of bleeding could not be demonstrated.^27,30^

### Neurodevelopmental outcome

We have not found an association between the Doppler-derived blood flow parameters and long-term neurodevelopmental outcome. However, this could be underpowered for methodological reasons, as the four studies included in this review used neither the same Doppler variables nor the same scale to evaluate neurodevelopmental outcome. The predictive capacity for long-term outcome of arterial Doppler-derived parameters investigated in past decades seems to be weak. Until now, it even remains unclear in which direction the association between Doppler parameters and outcome should be sought. An interesting hypothesis suggested by several authors is that low CBFV is a consequence rather than a cause of brain injury:^37^^,45^ the usual steady rise in CBFV caused by the increasing metabolic needs of the growing brain^61^ is lacking. The hypothesis is that a damaged brain does not grow as well as a normal brain and therefore demands less blood flow. The fact that lower brain volumes are associated with worse neurodevelopmental outcome supports this theory.^62^ To study predictability for outcome of arterial Doppler parameters, we should focus on studying the same artery and the same, standardized scale for neurodevelopmental outcome, which will allow to compare and pool results in future meta-analyses.

### General

In general, many factors are described to influence arterial cerebral blood flow, i.e., maternal smoking,^63^ being small for gestational age,^64^ premature rupture of membranes,^65^ pCO_2_,^66,67^ polycythemia,^68^ sedation,^69^ position of the baby,^70,71^ and hypoglycemia.^72^ The role of gestational age is less clear: some studies show a clear correlation with RI,^73^ whereas other studies show no difference.^47^ Many of the babies in the NICU are exposed to more than one of these factors, which may lead to inconsistent results or lack of significance.

Combining arterial cerebral blood flow parameters with clinical parameters or validated clinical scores (e.g., the SNAPPE-II scores) in a model would resolve this problem of significance. Combination of the clinical neurological exam with the existence of lesions on brain MRI or cranial ultrasound improved prediction of neurological outcome in preterm infants.^74^ As far as we know, no such relation was studied between cranial Doppler parameters in preterm babies and clinical parameters.

To the best of our knowledge, this is the first systematic review evaluating diagnostic and predictive value of commonly used cerebral blood flow Doppler-derived measures in relation to both short-term morbidity and longer-term outcome in preterm infants. Studies were published over a long period of time, going back to 1981, and in general had moderate or high risk of bias. We decided post hoc to exclude 15 studies that met all eligibility criteria because of a very high risk of bias in order to optimize the quality of our findings. One of the strengths of this systematic review is the fact that pre-specified inclusion and exclusion criteria addressing a specific review question were used. In addition, it included a comprehensive search, without limitation of year of publication, limiting risk of selection bias. Finally, formal assessment of risk of bias of included studies was performed allowing to weigh the strength of the evidence.

This review also has several limitations. The inclusion of older studies possibly reduced the applicability of our findings to current clinical practice due to the change in recent decades in the clinical characteristics of the preterm infants in a NICU and to the fact that some Doppler techniques are no longer in use. Also, older studies did not follow the strict standards of reporting that apply today and are therefore more prone to reporting bias. Another limitation of this study is the fact that quantitative analysis was not possible. This was due to important clinical and methodological heterogeneity. Studies varied considerably in terms of technique (duplex versus 2D ultrasound), Doppler parameters, insonated cerebral arteries, timing of measurement, and reporting (use of mean, median, 95% confidence interval, etc.).

It is possible that we did not find the appropriate Doppler parameter to approach cerebral blood flow yet. RI is often used, since this index shows a very small inter-observer variability and is not dependent on the angle of insonation. Reliability of other Doppler parameters, like end-diastolic, peak-systolic, and mean systolic velocities, is dependent on this angle between the flow velocity and the ultrasound beam. Modern ultrasound machines provide a “steer angle” tool to mathematically correct for this inaccuracy. However, calculations seldom provide true values. To bypass this constrain, using an index that is independent of this angle, like the RI, has become popular to approach CBFV. Greisen et al.^75^ showed that MV might be a better Doppler variable to assess cerebral blood flow patterns than RI, since the correlation coefficients with CBFV estimated by the Xenon-133 clearance technique were higher for MV than for RI. Unfortunately, in our review MV also showed a poor performance as predictive tool for IVH^41^ or PVL.^33,36^

An alternative method to approach cerebral circulation using Doppler ultrasound is the SVC flow,^76^ as 70–80% of the SVC flow represents the cerebral venous return. In several studies, an association between low SVC flow, impaired autoregulation, IVH, and adverse neurodevelopmental outcome was found.^14,15,41,77^ Unfortunately, some authors reported limited reproducibility of SVC flow measurement.^78,79^ However, with appropriate training functional echocardiography is becoming standard of care in many neonatal units in Australia, America, and Europe with reliable and reproducible results.^80,81^

The option of performing venous Doppler measurements instead of arterial ones could be interesting. The venous system is less influenced by vascular tone or shunts (i.e., PDA). With modern ultrasound technique and high-frequency probes, visualization of small vessels and very low flow states, like in the preterm venous circulation, is feasible, provided that the ultrasound probes are calibrated for the specific purpose.^82^ Measurements of changes in perfusion waveform in the internal cerebral vein showed a promising relation with IVH in extremely low birth weight infants.^83^ Future research could focus on the venous system of the preterm brain. Using sufficiently large populations, the same blood vessel at about the same anatomical place in the brain, and preferably in a (semi-)continuous monitoring mode could be a new approach of the cerebral blood flow of the preterm infant.

## Conclusion

According to this systematic review, there is no clear evidence to support the routine use of RI or other arterial Doppler-derived parameters to predict neurological outcome in the preterm infant. However, there is some evidence that elevated RI in the ACA and MCA can point to the presence of a hemodynamically significant PDA.

## Supplementary information

Appendix 1

Appendix 2
