# Supplementary material for: Diagnostic and predictive value of Doppler ultrasound for evaluation of the brain circulation in preterm infants: a systematic review
Source: Pediatr Res. 2020 Mar 26;87(Suppl 1):50–8. doi: 10.1038/s41390-020-0777-x (PMC7098887; doi:10.1038/s41390-020-0777-x)
Supplement: Supplementary file 1 — Appendix 1 [file 41390_2020_777_MOESM1_ESM.docx]

**General characteristics of included studies**

| **Reference + year (reference number)** | **Location** | **Aim** | **Design** | **Study population** | **Assessment parameter** | **Long-term Outcome** | **Comments** |
| --- | --- | --- | --- | --- | --- | --- | --- |
| Perlman  1981 (25) | Washington, USA | To investigate the effect of changes in the systemic circulation caused by PDA on blood flow velocity in the ACA. | Cohort with historical controls | Mean (range)  Preterm infants (n=55)  GA 30 wks (26-35)  BW 1160 g (650-2000) | Resistive Index (RI)  in ACA | No | Duplex flowmeter |
| Martin  1982 (26) | San Francisco, USA | To determine if retrograde flow occurs in cerebral arteries of infants who have retrograde flow in the descending aorta (due to PDA) | Cohort/ Convenience sample | Mean (± SD)  Large PDA group (n=7)  GA 29 wks (±2)  BW 1170 g (±300)  Small PDA group (n=3)  GA 30 wks (±3)  BW 1300 g (±130)  Control group (n=10)  GA 33 wks (±4)  BW 2050 g (±990) | Resistive Index (RI)  In ACA and MCA | No |  |
| Perlman  1982 (27) | Washington, USA | (1) to determine the effect of IVH on cerebral blood flow velocity  (2) to assess the reliability of the noninvasive Doppler technique in the diagnosis of IVH | Cohort with historical controls | Mean (range)  Infants with IVH (n=32)  GA 30 wks (26-34)  BW 1001g (680-1480) | Resistive Index (RI)  in ACA | No | Duplex flowmeter  Negative diastole defined as  PI = 1.0 |
| Ellison  1983 (28) | Milwaukee, USA | To compare cerebral blood flow parameters in infants with and without PDA | Case control/ convenience sample | Mean  Severe PDA group  GA 27 wks  BW 961 g  Mild PDA group  GA 27.3 wks  BW 948 g  Controls group  GA 30 wks  BW 1060 g | Resistive Index (RI)  and Mean flow velocity (MV)  in ACA and CCA | No | Duplex flowmeter |
| Deeg  1986 (29) | Erlangen, Germany | To determine the relationship between cerebral blood flow velocity and PDA | Case control/ convenience sample | Mean (± SD)?  PDA group (n=33)  GA 32 wks (±3)  BW 1268 g (±535)  Control group (n=96)  GA 37 wks (±4)  BW 2348 g (±944) | Resistive Index (RI)  End-diastolic velocity (Ved)  End-systolic velocity (Ves)  Maximum systolic velocity (Vs)  In ACA | No | Article in German |

| **Reference + year (reference number)** | **Location** | **Aim** | **Design** | **Study population** | **Assessment parameter** | **Long-term Outcome** | **Comments** |
| --- | --- | --- | --- | --- | --- | --- | --- |
| Van Bel  1987 (30) | Leiden,  The Netherlands | To eludicate the role of cerebral blood flow alterations on development of PIVH | Cohort | Mean (± SD)  Infants with PIVH (n=23)  - GA 28.7 wks (±2.2)  - BW 1246 g (±402)  Infants without PIVH (n=22)  - GA 30.0 wks (±1.9)  - BW 1592 g (±462) | Resistive Index (RI)  Area under the velocity curve (AUVC)  Coefficient of variation of PI and AUVC  In ACA | No | Duplex flowmeter |
| Kupferschmid 1988 (31) | Ulm, Germany | To study the diagnostic value of continuous wave Doppler compared to conventional m-mode cardiography and clinical parameters | Cohort | Median (range)  PDA group (n=29)  GA 29 wks (25-36)  BW 1030 g (470-2200)  Control group (n=29)  GA 29 wks (25-36)  BW 1040 g (470-2100) | Resistive Index (RI)  In ACA | No | Duplex flowmeter |
| Van Bel  1989 (32) | Leiden,  The Netherlands | To investigate whether the pattern of CBFV of preterm infants in the neonatal period is related to neurodevelopmental outcome | Case control/ convenience sample | Mean (± SD)  Normal infants (n=29)  - GA 30.0 wks (± 1.8)  - BW 1529 g (± 470)  Minor Impairment (n=11)  - GA 28.9 wks (± 2.4)  - BW 1208 g (± 356)  Major impairment (n=7)  - GA 29.4 wks (± 1.4)  - BW 1459 g (± 492) | Resistive Index (RI)  Area under the velocity curve (AUVC)  In ACA | Yes  - Touwen neuro-developmental screening  - Hearing  - Bayley Mental and Motor development score at 2 years corrected age. | Duplex flowmeter |
| Shortland  1990a (33) | Leicester,  UK | To assess the value of prospective measurements of cerebral and cardiovascular haemodynamics using Doppler ultrasound to determine whether it is possible to predict infants at risk of developing cerebral vascular pathology | Cohort | Median (IQR)  No PVH/PVL (n=65)  - GA 30 wks (28-32)  - BW 1.26 kg (1.04-1.47)  PVH (n=43)  - GA 28 wks (26-30)  - BW 1.13 kg (0.90-1.38)  PVL (n=7)  - GA 29 wks (27-30)  - BW 1.30 kg (1.08-1.52)  PVH + PVL (n=5)  - GA 28 wks (27-31)  - BW 1.10 kg (0.95-1.84) | Mean cerebral blood flow velocity  In ACA | No  Outcome = PVH of PVL | Duplex flowmeter |

| **Reference + year (reference number)** | **Location** | **Aim** | **Design** | **Study population** | **Assessment parameter** | **Long-term Outcome** | **Comments** |
| --- | --- | --- | --- | --- | --- | --- | --- |
| Shortland  1990 (34) | Leeds, UK | To determine whether infants who had patent ductus arteriosus were more likely to develop cerebral pathology, and to evaluate whether PDA had an effect on cerebral hemodynamics | Cohort | Median (IQR)  PDA group (n=34)  - GA 29 wks (IQR 28-32)  - BW 1240 g  Control group (n=70)  - GA 30 wks (IQR 28-32)  - BW 1140 g | Minimal, mean and maximal velocity  In ACA | No  Outcome = IVH and PVL | Relation between PDA and PVL/ IVH investigated, relation between PDA and CBFV investigated. Not directly between CBFV and outcome. |
| Mullaart 1994 (35) | Nijmegen, The Netherlands | To determine the relation between cerebral blood flow fluctuation and RDS and IVH | Cohort/ convenience sample | Range  Infants with PVH (n=8)  RDS without PVH (n=14)  Control group (n=13)  Total population  GA 26-35 weeks  BW 800-2030g | End diastolic velocity, mean velocity and peak systolic velocity  In right ICA | No  Outcome = PVH | Median velocity as measure for cerebral blood flow velocity  IQR as measure for cerebral blood flow fluctuation |
| Scherjon  1994 (36) | Amsterdam, The Netherlands | To report the relation of mean cerebral blood flow velocity in neonates to the occurrence of intracranial pathology and neurological outcome during the first year of life. | Cohort | Median (range)  Preterm infants (n=128)  GA 30 ^2^/­­_7_ wks (25 ^2^/_7_ - 32 ^6^/_7_)  BW 1280 g (605 – 2295) | Mean velocity and mean velocity ratio  In MCA | Yes  Prechtl score at 40 weeks GA  Touwen score at 6 and 12 months after birth. |  |
| Rennie  1995 (37) | Cambridge, UK | To compare Doppler measurement of cerebral blood flow velocity with real-time ultrasound imaging for the prediction of neurodevelopmental outcome at 18 months post-term. | Cohort | Mean (± SD)  Normal infants (n=42)  GA 29 wks (±2)  BW 1245 g (±217)  Delay/ handicap (n=9)  GA 27 wks (±1.3)  BW 1138 g (±263)  Died (n=15)  GA 26 wks (±1.6)  BW 924 g (±228) | Time-averaged velocity (TAV)  In ACA | Yes  Neurological examination and Bayley scales of infant development at 18 months corrected age |  |
| Coughtrey 1997 (38) | London, UK | To examine the variability in CBFV over 1 min, to compare this with variation in the systemic blood pressure, and to explore associations with intracranial injury and patent ductus arteriosus. | Cohort | Median (range)  Preterm infants (n=52)  GA 28.5 wks (24-32)  BW 1160g (501-1519) | Coefficient of variation  Area under the velocity curve (AUVC)  In “branches of the ACA and MCA” | No  Outcome = cerebral injury or death |  |

| **Reference + year (reference number)** | **Location** | **Aim** | **Design** | **Study population** | **Assessment parameter** | **Long-term Outcome** | **Comments** |
| --- | --- | --- | --- | --- | --- | --- | --- |
| Weir  1999 (39) | Ontario,  Canada | To examine the MCA blood flow velocity measured by pulsed Doppler in a cohort of ventilated, very low birth weight neonates with and without PDA. | Cohort | Mean (± SD)  Ventilated neonates < 1500g  PDA (n=43)  - GA 27.9 wks (± 1.8)  - BW 1071 (± 227)  No PDA (n=31)  - GA 27.4 wks (± 1.9)  - BW 1004 (± 192) | End-diastolic velocity, mean velocity, Resistive Index (RI) and Pulsatility Index (PI)  In right MCA | No |  |
| D’Orey  2000 (40) | Porto, Portugal | To evaluate arterial cerebral blood flow velocity changes in the presence of haemodynamically significant PDA and correlation with neurological morbidity and mortality of the preterm infant | Cohort | Mean (± SD)  PDA (n=15)  GA 27.8 wks (±2.2)  BW 1010.7 g (±290)  Control group (n=20)  GA 30.1 wks (±2.2)  BW 1330.3 g (±335) | Mean velocity (MV)  Resistive Index (RI)  In ACA, ICA and MCA | Partially  Primary outcome: IVH , PVL or death during NICU stay. Neurological outcome (Brunet-Lezine scale) at 12 months briefly mentioned (n=24) |  |
| Evans  2002 (41) | Sydney, Australia | To relate the postnatal changes in cerebral Doppler velocity indices to subsequent cerebral morbidity | Cohort | Mean (range)  Preterm infants < 30 wks (n=126)  GA 27 wks (23-29)  BW 991g (420-1630) | Systolic and diastolic velocity, mean velocity (MV), Pulsatility Index (PI)  in ACA and MCA  +  Area under velocity curve in MCA | No  Outcome = IVH |  |
| Okumura  2002 (42) | Aichi,  Japan | To prospectively elucidate the relation among cerebral bloodflow, periventricular Leukomalacia and hypocarbia using Doppler ultrasonography. | Cohort | Mean (± SD)  PVL group (n=12)  GA 30.1 wks (± 1.9)  BW 1523 g (± 387)  Control group (n=41)  GA 30.1 wks (± 2.4)  BW 1464 g (± 419) | Mean velocity and Resistive Index (RI) | Yes  Primary outcomes:  1. PVL at term age  2. Follow-up 2 years |  |
| Ojala  2004 (43) | Turku, Finland | To evaluate the relation between cerebral blood flow un the first day of life and the subsequent psychomotor development at the corrected age of 1 year in preterm infants. | Cohort | Mean (SEM)  Ventilated infants (n=35)  GA 29 wks (0.6)  BW 1258g (476)  Non-ventilated (n=14)  GA 31 wks (0.6)  BW 1719g (334) | Cerebral blood flow velocity, cerebral blood flow resistance and cerebrovascular perfusion pressure  In ACA | Yes  Griffith’s mental developmental scale at 12 months corrected age |  |

| **Reference + year (reference number)** | **Location** | **Aim** | **Design** | **Study population** | **Assessment parameter** | **Long-term Outcome** | **Comments** |
| --- | --- | --- | --- | --- | --- | --- | --- |
| Jim  2005 (44) | Taipei, Taiwan | To assess the hemodynamics of the cerebral arteries and occurrence of intraventricular hemorrhage in very-low-birth weight infants with and without patent ductus arteriosus. | Cohort | Mean (± SD)  Significant PDA (n=40)  GA 27.7 wks (±2.6)  BW 1063.3 g (±277.0)  Control group (n=37)  GA 28.7 wks (±2.7)  BW 1113.0 g (±189.3) | Resistive Index (RI)  In ACA | No  Outcome = IVH |  |
| Fukuda  2006 (45) | Nagoya, Japan | To investigate the developmental changes in blood flow in each cerebral artery among infants with and without periventricular Leukomalacia, to elucidate the time of onset of PVL. | Cohort | Mean (± SD)  Cystic PVL (n=8)  GA 30.6 wks (±17d)  BW 1351 g (±335)  Control group (n=59)  GA 28.4 wks (±20d)  BW 1191 g (±392) | Mean cerebral blood flow velocity (Mean CBFV)  In ACA, MCA, PCA, ICA and Basilar artery | Yes, partially  Primary outcome = Cystic PVL at day 21 or day 28  Secondary outcome = Cerebral Palsy at 12 months of age |  |
| Brissaud 2012 (46) | Bordeaux, France | To study the predictive value of ultrasound morphological and hemodynamic abnormalities for a severe adverse neurological outcome | Cohort | Median (IQR)  Preterm infants <33 wks (n=452)  GA mean (24-32 wks)  BW 1320g (1030-1630) | Resistive Index (RI)  Peak systolic bloodflow velocity  in ACA | Severe adverse neurological outcome at hospital discharge |  |
| Ecury-Goossen 2016 (47) | Rotterdam, The Netherlands | To compare resistive index in preterm infants <29 weeks gestation in various cerebral arteries and assess the relationship between resistive index and haemodynamically significant patent ductus arteriosus | Cohort | Median (range)  Preterm infants < 29 wks (n=235)  GA 27 wks (23^6^/_7_-28^6^/_7_)  BW 920g (360-1610) | Resistive Index (RI)  In ACA, ICA, Basilar Artery, Striatal arteries and Pial arteries | No |  |

GA – Gestational Age BW – Birth Weight IQR – Interquartile Range PDA – Patent Ductus Arteriosus

ACA – Anterior Cerebral Artery MCA – Middle Cerebral Artery CCA – Common Carotid Artery PCA – Posterior Cerebral Artery

ICA – Internal Carotid Artery IVH – Intraventricular Hemorrhage PIVH – Peri-Intraventricular Hemorrhage PVH – Periventricular Hemorrhage

PVL – Periventricular Leukomalacia CBFV – Cerebral Blood Flow Velocity RDS – Respiratory Distress Syndrome LA:AO ratio – Ratio between left atrial diameter and aorta diameter.
