# Supplementary material for: Diagnostic and predictive value of Doppler ultrasound for evaluation of the brain circulation in preterm infants: a systematic review
Source: Pediatr Res. 2020 Mar 26;87(Suppl 1):50–8. doi: 10.1038/s41390-020-0777-x (PMC7098887; doi:10.1038/s41390-020-0777-x)
Supplement: Supplementary file 2 — Appendix 2 [file 41390_2020_777_MOESM2_ESM.docx]

**Summary of findings of included studies**

| **Reference + year (reference number)** | **Risk of bias** | **Relevant results in outcome group** | **Results in comparison group** | **Main findings** | **Comments** |
| --- | --- | --- | --- | --- | --- |
| Perlman  1981 (25) | High | RI - Mean (95% CI*)  PDA 0.91 (0.89-0.93) | RI - Mean (95% CI*)  Controls 0.66 (0.64-0.68) | - Resistive Index in ACA increased in hemodynamic significant PDA (p<0.001)  - Lower RI in PDA mainly caused by lower diastole | Duplex flowmeter |
| Martin  1982 (26) | High | RI Mean (95% CI*)  Large PDA 0.96 (0.92-1.0) | RI Mean (95% CI*)  Controls 0.75 (0.73-0.77) | - Resistive Index in ACA increased in large PDA (p<0.001)  - In small PDA is RI comparable to controls. | Duplex flowmeter  In case of 0-flow or negative diastole, defined as RI = 1.0 |
| Perlman  1982 (27) | High | RI - Mean (95% CI*)  IVH  Day 1-5 0.59 (0.57-0.61) | RI - Mean (95% CI*)  Controls  First month 0.66 (0.64-0.68) | - No consistent relationship IVH and either the absolute values of Resistive Index in the ACA or the changes in RI after the diagnosis of IVH. | Duplex flowmeter |
| Ellison  1983 (28) | High | Mean RI, mean MV in cm/s  In severe PDA (5 days before ligation):  * ACA PI 0.78, MV 6.37  * CCA PI 0.90, MV 4.28 | Mean RI, mean MV in cm/s  Controls  * ACA PI 0.61, MV 9.22  * CCA PI 0.84, MV 7.80 | - No difference in first 4 days of life  - 5 days before surgical ligation: Significant higher Resistive Index in ACA (p<0.01) and significant lower MV in both ACA (p=0.04) and CCA (p<0.01) in severe PDA. | Duplex flowmeter |
| Deeg  1986 (29) | High | Mean velocity in cm/s (95% CI*),  RI mean (95% CI*)  Large PDA  Vps 34 (30.5 - 37.6)  Ves 4 (2.2 - 5.8)  Ved - 4 (-5.8 - -2.2)  RI 1.11 (1.06 – 1.16) | Mean velocity in cm/s (95% CI*),  RI mean (95% CI*)  Controls  Vps 41 (38.6 - 43.4)  Ves 19 (17.4 - 20.4)  Ved 10 (9.2 - 10.8)  RI 0.75 (0.73-0.77) | - Overall lower velocities in ACA in large PDA  (Vps p<0.01, Ves p<0.0001, Ved p<0.0001\|)  - Resistive Index elevated in large PDA and slightly elevated in small PDA (p<0.0001, p <0.0001) | In negative diastolic flow 🡪 RI > 1.0 |
| Van Bel  1987 (30) | Low | RI, AUVC in cm/min,  Variation RI and variation AUVC in%  - Mean (95% CI*)  Severe PIVH  RI 32h 0.36 (0.32-0.39)  40h 0.39 (0.32-0.46)  64h 0.41 (0.34-0.48)  AUVC 40h 684 (507-861)  Var PI 32h 33 (20.7-45.3)  48h 25 (18.1-31.9)  64h 24 (14.0-34.0)  Var AUVC 32h 21 (7.9-34.1)  64h 13 (8.4-17.6)  96h 13 (10.7-15.3)  108h 12 (9.7-14.3) | RI, AUVC in cm/min,  Variation RI and variation AUVC in%  - Mean (95% CI*)  Non-PIVH  RI 32h 0.46 (0.43-0.49)  40h 0.46 (0.42-0.49)  64h 0.49 (0.45-0.53)  AUVC 40h 645 (568-722)  Var PI 32h 13 (10.8-15.2)  48h 12 (10.2-13.7)  64h 11 (9.3-12.7)  Var AUVC 32h 10 (7.8-12.2)  64h 10 (8.7-11.3)  96h 10 (8.7-11.3)  108h 8 (6.7-9.3) | - First 24 hours no difference between cerebral blood flow in ACA in infants who develop severe PIVH compared to non-PIVH infants.  - In severe PIVH:  * Resistive Index in ACA is lower at 32, 40 and 64h of age (p<0.01, p<0.05 and p<0.01 respectively)  * AUVC in ACA higher at 40 hours of age (p<0.05)  * Coefficient of variation RI higher at 32, 48 and 64 hours of age (p<0.001, p<0.01 and p<0.001 resp.)  * Coefficient of variation AUVC higher at 32, 64, 96 and 108 hours of age (p<0.05)  - No relation between RI in ACA and time of onset of PIVH.  - Significant correlation between variation of RI and time of onset of PIVH (p<0.01). | Duplex flowmeter |
| Kupferschmid 1988 (31) | High | RI Median (range)  PDA 1.0 (1.0-1.2) | RI Median (range)  Controls 0.68 (0.58-0.86) | - Absent or retrograde diastolic blood flow in the ACA (Resistive Index ≥ 1.0) is the most sensitive (100%) and specific (100%) test to diagnose haemodynamically significant PDA in preterm infants compared to clinical findings and M-mode cardiography. | Duplex flowmeter |

| **Reference + year (reference number)** | **Risk of bias** | **Relevant results in outcome group** | **Results in comparison group** | **Main findings** | **Comments** |
| --- | --- | --- | --- | --- | --- |
| Van Bel  1989 (32) | Moderate | RI - Mean (95% CI*)  Severe Handicap  48h 0.50 (0.42-0.57)  64h 0.52 (0.49-0.55)  72h 0.58 (0.52-0.64)  120h 0.57 (0.48-0.66)  132h 0.60 (0.54-0.66)  156h 0.62 (0.57-0.67) | RI - Mean (95% CI*)  Normal  48h 0.44 (0.41-0.47)  64h 0.45 (0.42-0.48)  72h 0.47 (0.44-0.50)  120h 0.48 (0.44-0.52)  132h 0.50 (0.48-0.52)  156h 0.51 (0.48-0.54) | - Infants with severe impairment show higher Resistive Index values in the ACA in the first week of life  (significant at 48^1^, 64^1^, 72^3^, 120^1^, 132^2^ and 156^3^ hours; ^1^p<0.05, ^2^p<0.02, ^3^p<0.001) | Duplex flowmeter |
| Shortland  1990a (33) | High | Results presented in table | Results presented in table | - No statistically significant differences in CBFV in the ACA in infants that will develop PVH, PVL or both compared to normal infants in the first 7 days of life. | Duplex flowmeter  Infants excluded after developing first lesion |
| Shortland  1990 (34) | Moderate | Minimum CBFV in cm/s  PDA  D3 0.13 | Minimum CBFV in cm/s  PDA  D3 2.88 | - No significant differences in CBFV in ACA in infants with and without PDA, except for the minimum CBFV on day 3 (p=0.02).  - Infants with PDA are more likely to develop cerebral ischemic lesions than infants without PDA (p<0.02). No association between PDA and PVH.  - Infants with end-diastolic retrograde flow in the ACA are more likely to develop PVL (+/- PVH) than infants without retrograde flow (36% versus 15%, p<0.02) | No direct association between CBFV and outcome |
| Mullaart  1994 (35) | High | Mean cerebral blood flow fluctuation in cm/s (90% intervals range)  PVH 0.7 – 2.6  RDS without PVH 0.6 – 2.2 | Mean cerebral blood flow fluctuation in cm/s (90% intervals range)  Controls 0.6 – 2.3 | - A relationship between PVH and CBFF exists, but seems to be almost completely explained by PDA and RDS. | Interquartile range of velocity = measure of cerebral blood flow fluctuation |
| Scherjon  1994 (36) | Low | MV, Min-max MV-ratio and Vmean-ratio – only statistics given, no absolute values | Normal MV pattern in first week of life presented in figure.  Min-max MV-ratio – median (range) in entire study population 0.64 (0.25-1.24) | - Neither absolute MV values nor MV ratio in MCA were related to ultrasound findings (PIVH, PVL).  - Vmean-ratio was associated with Prechtl-score (p=0.009) at term age. No relation with Touwen’s neurological examination at 6 or 12 months.  - Neither absolute values of MV nor Min-max VM-ratio were related to abnormal outcome according to Prechtl’s or Touwen’s neurological examination. | V mean-ratio =  MV in first 12 h/MV at 12-168h  Min-max MV-ratio =  Minimal MV/ maximal MV |
| Rennie  1995 (37) | Low | Mean TAV in cm/s (95% CI*)  Impaired infants  D1 4.25 (3.3-5.2)  D2 5.7 (4.3-7.1)  D3 5.5 (4.0-7.0) | Mean TAV in cm/s (95% CI*)  Normal infants  D1 4.3 (3.7-4.9)  D2 6.5 (5.7-7.3)  D3 6.5 (5.9-7.1) | - No significant differences in mean CBFV in ACA between normal and impaired infants.  - Infants with abnormal neurological signs and developmental delay did not show the usual steady rise in CBFV in ACA during the first few days of life.  - Structural abnormality seen on the cranial ultrasound image was a better predictor of adverse outcome than an abnormal CBFV pattern in ACA. |  |

| **Reference + year (reference number)** | **Risk of bias** | **Relevant results in outcome group** | **Results in comparison group** | **Main findings** | **Comments** |
| --- | --- | --- | --- | --- | --- |
| Coughtrey 1997 (38) | High | Median maximum coefficient of variability in % (IQR)  Babies who died 31 (20-38)  Babies with cerebral injury 28 (10-30)  Babies surviving intact 13 (10-30) | Variability of CBFV in median % (IQR)  Normal 8 (2-28%) | - Higher coefficient of variability in CBFV in ACA in hypotension.  - Strong correlation between hypotension on day 1 and cerebral injury (p=0.006)  - Babies who died had a higher maximum coefficient of variation (p=0.05)  - No association confirmed between coefficient of variation and brain injury or PDA. | Results presented in figure |
| Weir  1999 (39) | Moderate | Only p-values and regression equations given, no absolute values | Only p-values and regression equations given, no absolute values | - PDA significantly reduced MV (p=0.129), reduced end-diastolic velocity (p=0.008) and increased Resistive Index (p=0.047) in the MCA in the first 5 days of life.  - No significant effect of PDA found on peak-systolic velocity or PI. |  |
| D’Orey  2000 (40) | High | Mean MV in cm/s (± SE) and RI (± SE)  In large PDA  MV in ACA 30.2 (± 1.4)  MCA 37.4 (±2.6)  ICA 40.5 (± 2.6)  RI in ACA 0.94 (± 0.2)  MCA 0.94 (± 0.2)  ICA 0.97 (± 0.3) | Mean MV in cm/s (± SE) and RI (± SE)  In control group  MV in ACA 34.9 (± 1.3)  MCA 41.6 (±2.3)  ICA 45.8 (± 2.2)  RI in ACA 0.81 (± 0.2)  MCA 0.80 (± 0.2)  ICA 0.81 (± 0.2) | - Significant reduction in average velocity, end-diastolic velocity and high Resistive Index in ACA, MCA and ICA in very low birth weight infants with a large PDA (p<0.05) |  |
| Evans (41)  2002 | Low | MV in m/s, PI – Median (range)  Late grade 1-4 IVH  Minimum MV 0.095 (0.03-0.16)  Maximal PI 1.96 (1.33-4.28) | MV in m/s, PI – Median (range)  No IVH  Minimum MV 0.11 (0.02-0.29)  Maximal PI 1.84 (0.77-12.3) | - No significant association between minimum MV (p=0.18) and maximal Pulsatility Index (p=0.78) in MCA between 5 and 12 hours of life and late IVH.  - Positive association between SVC-flow and IVH (P<0.0001) |  |
| Okumura  2002 (42) | High | RI, MV in cm/s – Mean (± SEM)  PVL  MV at D1 14.4 (±1.2)  D3 17.0 (±1.4)  RI at D1 0.62 (± 0.022),  D3 0.60 (± 0.032) | RI, MV in cm/s – Mean (± SEM)  No PVL  MV at D1 15.0 (±0.72)  D3 15.0 (±0.80)  RI at D1 0.71 (± 0.014),  D3 0.66 (± 0.013) | - Reduced Resistive Index in ACA in infants with PVL during the first 72 hours of life (D1 p<0.01, D3 p<0.05).  - No differences in MV in infants with and without PVL  - RI or MV not different regardless of the presence or absence of hypocarbia |  |
| Ojala  2004 (43) | Moderate | Results presented in figure | Results presented in figure | - In the whole study population CBFV, cerebrovascular perfusion pressure and CBF resistance were associated with the sum of Griffith’s developmental scales (p<0.02)  - The correlations between CBF and CBF resistance and the sum of Griffith’s developmental scales were significant only in non-ventilated preterm infants p=0.07 and p=0.001 respectively).  - For the cerebrovascular perfusion pressure this correlation existed for both ventilated and non-ventilated infants. |  |

| **Reference + year (reference number)** | **Risk of bias** | **Relevant results in outcome group** | **Results in comparison group** | **Main findings** | **Comments** |
| --- | --- | --- | --- | --- | --- |
| Jim  2005 (44) | Low | RI results presented in figure | RI results presented in figure | - Significant positive linear relation between LA:AO ratio and Resistive Index (p<0.001), and inverse linear relation with Vd (p=0.02).  - Overall incidence of IVH significantly higher in PDA-group (p=0.006), however there was no statistical difference in the grading or severity of IVH. | No direct correlation between RI and IVH (only between RI and LA:AO ratio and between PDA and IVH) |
| Fukuda  2006 (45) | Moderate | Results presented in table | Results presented in table | - Mean CBFV of infants with PVL is lower in all investigated arteries on day 0 (NS)  - Mean CBFV of infants with PVL is significantly lower in the PCA and ICA after the first week of life and in the ACA, MCA and basilar artery after the second week of life (p<0.05) | No analysis done to examine relation between CBFV and cerebral palsy |
| Brissaud  2012 (46) | Low | Peak systolic velocity Vps in cm/s  Infants with Severe Adverse Neurological Outcome (SANO)  Vps 33.8 cm/s  Velocity <25 cm/s in 21.5% of infants | Control group  Vps 35.8 cm/s  Velocity <25 cm/s in 12.7% of infants | - Early velocity measurements (day 3-8) in ACA tend to be lower in children at risk for late severe ultrasound abnormality or neurological related death (p=0.12 for Vps; p=0.06 for % of values with Vps <25 cm/s)).  - No association between early RI (day 3-8) and SANO (p=0.89)  - Early ultrasound abnormality strongest predictor of a further SANO. |  |
| Ecury-Goossen 2016 (47) | Low | RI median (IQR)  Haemodynamically significant PDA  ICA right 0.81 (0.75-0.87)  ICA left 0.85 (0.80-0.90)  ACA 0.82 (0.78-0.87) | RI median (IQR)  No PDA  ICA right 0.79 (0.73-0.84)  ICA left 0.79 (0.75-0.84)  ACA 0.75 (0.70-0.81) | - Resistive Index in large arteries consistently higher compared to smaller arteries. Therefore, for serial assessment of RI, measurements must be made in the same artery at roughly the same place.  - Significant association between PDA and RI in ACA and left ICA (both p<0.001) | RI in left ICA significantly higher in PDA, no significance in right ICA, although differences are *clinically* irrelevant. Authors state that asymmetry might be explained by ductal steal phenomenon or measurement variability. |

SANO - severe adverse neurological outcome Vps – Peak-systolic velocity RI – Resistency Index (= Pourcelot Index) ACA – Anterior Cerebral Artery

IQR – Interquartile Range CBFV – Cerebral Blood Flow Velocity PDA – Patent Ductus Arteriosus MV - Mean velocity

SE - Standard Error MCA – Middle Cerebral Artery ICA – Internal Carotid Artery Ves - End-systolic velocity

Ved – End-diastolic velocity CCA – Common Carotid Artery IVH – Intraventricular Hemorrhage SVC-flow – Superior Vena Cava flow

PVL – Periventricular Leukomalacia PCA – Posterior Cerebral Artery Vd - diastolic velocity LA:AO ratio – Ratio between left atrial diameter and aorta diameter

PVH – Periventricular Hemorrhage RDS – Respiratory Distress Syndrome CBF – Cerebral Blood Flow SEM – Standard Error of Means

TAV – Time Averaged Velocity PIVH – Peri-Intraventricular Hemorrhage AUVC – Area Under the Velocity Curve Var - variation

* 95% Confidence Interval calculated from data
